# Supplementary figures and images for: Novel measurement tool and model for aberrant urinary stream in 3D printed urethras derived from human tissue
Source: PLoS One. 2020 Nov 11;15(11):e0241507. doi: 10.1371/journal.pone.0241507 (PMC7657556; doi:10.1371/journal.pone.0241507)

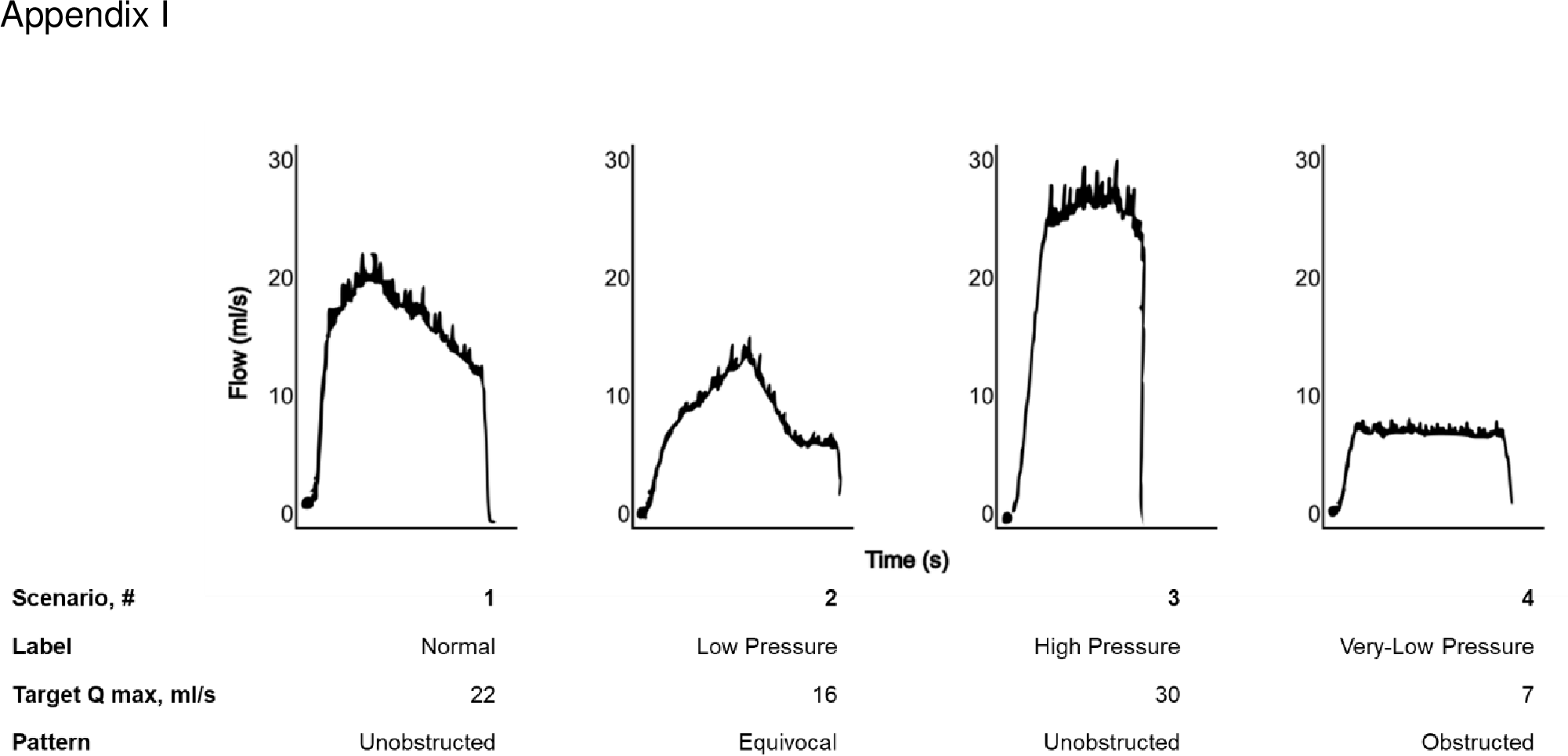

Supplement: S1 Appendix — (TIF) [file pone.0241507.s001.tif]
