# Supplementary material for: Novel measurement tool and model for aberrant urinary stream in 3D printed urethras derived from human tissue
Source: PLoS One. 2020 Nov 11;15(11):e0241507. doi: 10.1371/journal.pone.0241507 (PMC7657556; doi:10.1371/journal.pone.0241507)
Supplement: Attachment — Submitted filename: PLOS response2.docx [file pone.0241507.s004.docx]

**Please use the space provided to explain your answers to the questions above. You may also include additional comments for the author, including concerns about dual publication, research ethics, or publication ethics. (Please upload your review as an attachment if it exceeds 20,000 characters)**

**Reviewer #1: Thank you for carefully considering and addressing the comments made. There are just some small remaining issues to sort:

You use 'dispensability' twice now on p.12, whereas I think you mean 'distensibility'.

In the new text on p.12, you use 'recapitulate' which may be a valid technical term in modelling, but it does not communicate well here. I suggest 'did not reproduce live human tissue' is clearer, as you have done in the abstract.**

Thank you again for your helpful comments and keen eye for errors. We have made these corrections on page 12 .

**Your comment on shear wave elastography is interesting, and you may be interested to check out J Urol 2017; 198:422-429. I agree that this method may well be worth future investigation.

In Tables 1 and 2, the addition of horizontal and vertical lines may assist understanding. I found it not immediately obvious which figures the p-values corresponded with, and also where the division between specimens lay.**

We have added some vertical and horizontal lines to aid in drawing the readers eye. We are open to any and all table formatting suggestions to bring the paper in line with PLOSone formatting standards without reservation.

**Reviewer #2: Discussion, first paragraph: intrauthreal should be corrected.**

Thank you, this spelling error corrected.

**In the Discussion, more attention is paid now to the differences between living tissue, cadaveric urethras and 3D models, that is, to the limitations of the study. There are large differences between results obtained in the cadaveric urethras and the 3D models. Nevertheless, this study seems a good onset of a series of experiments and observations on the value of 3D models in clinical practice.**
